# Synthesis and Formulation of Thermosensitive Drug Carrier for Temperature Triggered Delivery of Naproxen Sodium

**DOI:** 10.3390/molecules21111473

**Published:** 2016-11-04

**Authors:** Monika Gasztych, Agnieszka Gola, Justyna Kobryń, Witold Musiał

**Affiliations:** Department of Physical Chemistry, Pharmaceutical Faculty, Wroclaw Medical University, Borowska 211, Wroclaw 50-556, Poland; monika.gasztych@umed.wroc.pl (M.G.); agnieszka.gola@umed.wroc.pl (A.G.); justyna.kobryn@umed.wroc.pl (J.K.)

**Keywords:** *N*-isopropylacrylamide, hydrogel, naproxen sodium, polymerization, drug release

## Abstract

Nanospheres and microspheres are known as a multipurpose compounds and are used in various branches of science. Recent controlled delivery systems for drugs are also based on poly-micro and nanospheres. In our study we describe an investigation of the influence of thermosensitive polymer *N*-isopropylacrylamide (NIPA) on the release of the drug naproxen sodium (NS) with a hydrogel hydroxypropyl methylcellulose (HPMC) base. The hydrodynamic diameter (D_H_) of the obtained polymer was measured by using dynamic light scattering (DLS) at a wavelength of 678 nm. Hydrogel formulations of NS were prepared in a specific way ex tempore. NS was sprinkled on the surface of a distilled water, then polymer soluted in water was added. Afterward, HPMC was affixed to the solution. Prepared samples were stored at room temperature for 24 h. Release tests showed that modification of thevcross-linker type influenced the properties of synthesized polymeric particles. The NIPA derivatives obtained via surfactant free precipitation polymerization (SFPP) may be formulated as hydrogel preparations using HPMC. The obtained formulations presented varied half-release times, depending on the type of applied NIPA derivatives in hydrogel formulations. At 18 °C, the release rates were lower comparing to the reference HPMC hydrogel, whereas at 42 °C, the release rates were significantly higher. The synthesized thermosensitive polymers enabled temperature-triggered release of NS.

## 1. Introduction

Nanospheres and microspheres are common in the scientific world, and their reception and use have improved over the last few decades [1]. Controlled drug delivery systems have been attracting attention for many years. Reducing the dosing frequency to mitigate the side effects of medication make this system very interesting and desirable [2]. Moreover, it provides long and stable therapeutic effects and protects the drug substance from rapid degradation. The choice of materials for the drug’s form is very important, with regard to their chemical and physical properties. Their sensitivity to an organism’s physiological conditions (e.g., temperature, pH) makes the controlled release process active. The release rate of the drug from the nano and microspheres depends on many factors and properties of the active substance, such as content and particle size. The properties of a polymer—a reserved substance—also plays a significant role. It is possible to change the release process and to increase the stability of the substance by modifying these parameters [3]. Hydrophilic hydrogels, which exhibit a lot of unique physicochemical properties, are used for biomedical applications, for example to deliver drugs. Mathematical models play an important role in facilitating the design of hydrogels by identifying the most important parameters [4,5,6]. Hydrogels can be prepared from natural and synthetic polymers. However, those made from natural polymers may not provide sufficient mechanical properties, and may contain infectious agents and induce inflammatory reactions. On the other hand they have advantages such as biocompatibility and biodegradability. Synthetic polymers are not bioactive. However, they usually have well-defined structures that can be modified to provide biodegradability [4]. Hydrogels are an excellent material to encapsulate the biological macromolecules, for example proteins or DNA, due to the fact that they are devoid of hydrophobic interactions which might cause denaturation [4]. Polymeric hydrogels, also known as “intelligent hydrogels”, pass transitions phase at the action of external stimuli, such as temperature and pH [3,7]. That kind of conditions may make them swollen [1,8]. Poly(*N*-isopropylacrylamide) (NIPA) is a representative substance sensitive to temperature as well as the main co-monomer used in the presented studies [9,10]. Its lower critical solution temperature (LCST) is 32−34 °C, and the volume phase transition temperature (VPTT) is close to the physiological temperature [11,12]. Although the first synthesis of NIPA was in 1986 by Pelton and Chibante, the polymer is still appealing. NIPA is made of hydrophilic amide groups and also of hydrophobic isopropyl groups [13,14]. It is water-soluble because of hydrogen bonds in the central amide group which rival with the hydrophobic fragments of the chain [15,16]. In an aqueous environment, NIPA below the LCST is soluble and hydrophilic, and above this temperature is hydrophobic and undergoes precipitation, becoming a cloudy dispersion [17,18,19]. It is possible to affect the value of LCST by copolymerizing hydrophobic monomers or by controlling the molecular weight of the polymer [20,21]. The hydrophobic monomers and their high molecular weight reduce the value of LCST, and the hydrophilic monomers which create hydrogen bonds with the thermo-sensitive monomers increase the LCST. Due to these modifications, we can get a different VPTT for NIPA copolymers [17]. NIPA and its derivatives are great for creating thermo-sensitive structures. Modifications of LCST affect the drug release rate and can largely improve the therapeutic efficacy of biomolecules [1,5]. NIPA is tolerated well by organisms, and therefore there are possibilities for using it in medicine and biotechnology [22,23]. In the previous scientific work for synthesis potassium persulfate (KPS) was used as an initiator. It is the initiator which forms terminal anionic groups with an acidic nature [24].

The aim of the study was to investigate the effect of the sensitive NIPA for the release of the drug substance naproxen sodium (NS) with hydrogels based on hydroxypropyl methyl cellulose (HPMC).

## 2. Results

### 2.1. NMR Spectra

**Figure 1:**
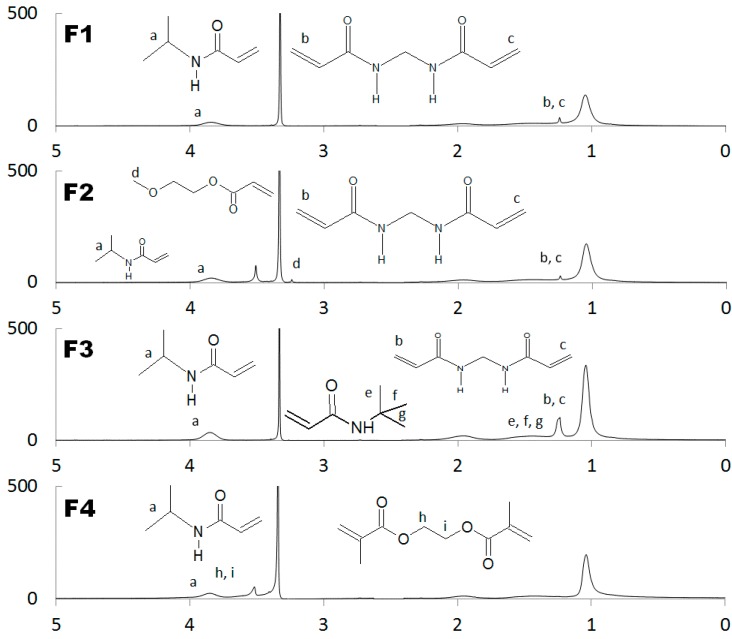
^1^H-NMR spectra of respective synthesized polymers F1–F4.

In order to determine the specific chemical structure of the synthesized polymers we carried out NMR analysis of the resulting compounds and all the components used in the synthesis. Information about the structure of synthesized NIPA derivatives was evaluated from the number of signals indicative for different kinds of protons in the molecule; position and intensity of signals was determined. The course of the polymerization reaction was clearly visible, as in all of the products (F1–F4, Figure 1) signals from the protons linked to the -C=CH bond present in the spectra of the monomer at 5.5–6.7 ppm disappeared. This confirms the absence of unreacted raw materials—monomers, oligomers, and co-monomers—in obtained products. In all NMR spectra of the obtained products the signals from protons observed around 3.85 ppm indicated the presence of a tertiary isopropyl group—δ 3.82 (1H, septet). The signals in the 1.25 ppm range confirmed the presence of protons of methylene groups in saturated *N*,*N*’-methylene bisacrylamide (MBA): δ 1.25 (3H, d), polymers F1–F3 on Figure 1. F2 polymer on the Figure 1 reveals signals of methoxy group—δ 3.34 (4H, s). Also, the incorporation of *N*-tertbutyl acrylamide (NTB) into the polymer was confirmed via the signals at 1.32 ppm, indicating presence of respective methylene group: δ 1.32 (1H, s)—F3 on Figure 1. The signals from the poly (ethylene glycol) dimethacrylate (PEG-DMA) were found in the specter of F4: δ 3.5–4.0 (multiplet) according to the identified ethylene group.

### 2.2. Hydrodynamic Diameter and VPTT Evaluations

The results of D_H_ at 22 °C and 42 °C as well as VPTT obtained from continuous hydrodynamic diameter (D_H_) measurements are presented in Table 1. F1 and F3 polymers were characterized by a distinct VPTT value; however, we failed to clearly define the VPTT in the case of products F2 and F4. In the case of F2 and F4 there was no clear point drop on the plot of D_H_ against temperature in the range of 22–42 °C.

### 2.3. Release Kinetics Evaluated due to Selected Kinetic Models

**Figure 2:**
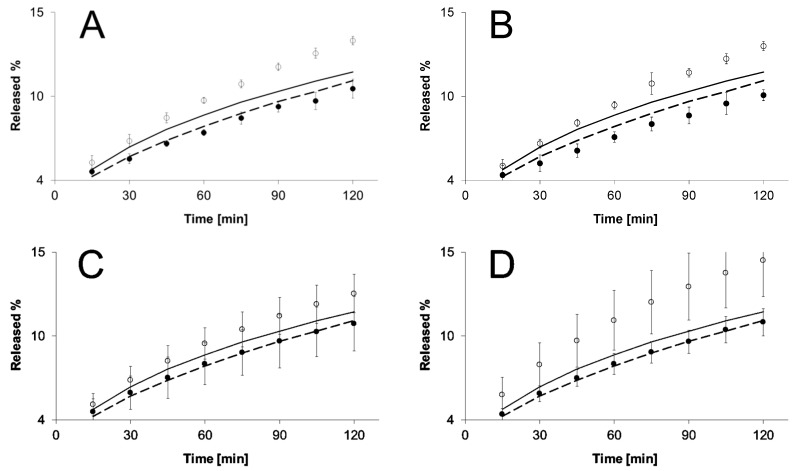
Release of NS from the formulations FP1 (**A**); FP2 (**B**); FP3 (**C**); FP4 (**D**) at 22 °C (●) and 42 °C (○) containing the thermosensitive polymers, the lines reflect release of NS from the HPMC reference gel (REF) at 22 °C (dotted, ---), and at 42 °C (solid line, **-**). The SD did not exceed 0.17 in the case of REF at 22 °C, and 0.93 at 42 °C.

Release courses of NS at 22 °C for all assessed formulations were similar to each other and also in comparison to the control formulation. However, following the temperature increase to 42 °C, there was a clearly faster release rate of the drug substance. In formulations FP1–FP3, results were similar and were higher compared to the control formulation without thermosensitive polymer. In the case of formulation FP4, the highest percentage of released NS was observed at 42 °C. Specific differences between formulations FP1–FP4 releasing NS at different temperatures are presented in Figure 2.

The results of release rate assays calculated according to selected kinetic models are presented in Table 2 and Table 3.

**Figure 3:**
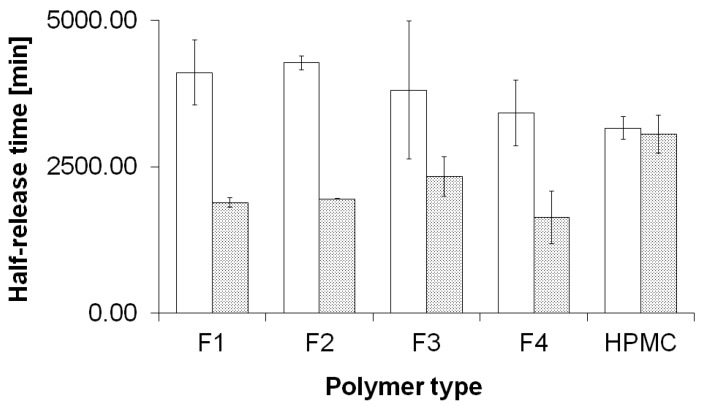
Half-release times for the NS released from the hydrogels based on thermosensitives polymers F1–F4 compared to HPMC hydrogel, at 22 °C (white columns), and at 42 °C (grey columns).

We included in the tables the respective release rates for zero-order, first-order, second-order and Higuchi kinetics equations with regression coefficients for evaluated kinetic models. For the FP1 in 22 °C the zero order rate constant is 5.18 × 10^−2^% × min^−1^ but in 42 °C it was 7.59 × 10^−2^% × min^−1^. For the reference formulation there is the same trend: in 22 °C the value is lower, 5.86 × 10^−2^% × min^−1^, than in 42 °C, 5.93 × 10^−2^% × min^−1^. For the first order process the highest value is presented by the FP4 in both temperatures: in 22 °C the rate constant is 6.11 × 10^−4^ min^−1^ and in 42 °C it is 9.18 × 10^−4^ min^−1^. The respective half-release times are depicted in Figure 3.

## 3. Discussion

**Figure 4:**
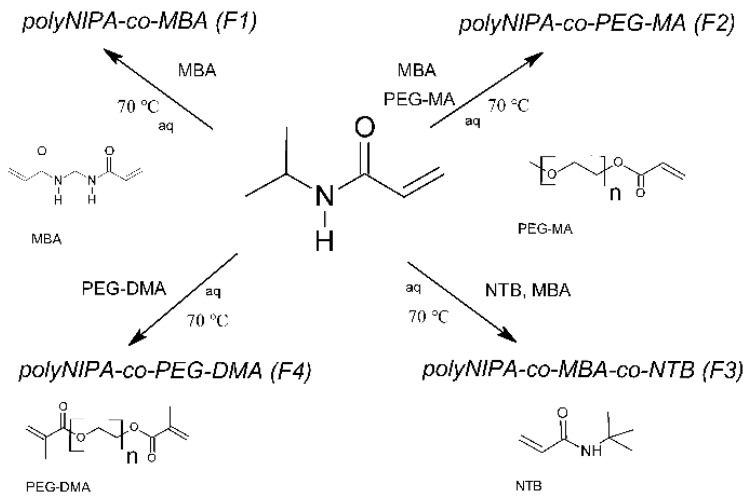
Schematic course of performed SFPPs of particles F1–F4.

According to the available data, the SFPP results in particles of spherical shape, whereas the cationic initiator provides a positive charge on the particle surface. Using of methyl ether-acrylate poly (ethylene glycol) (PEG-MA) in the preparation F2 resulted in microspheres, characterized by a high D_H_ 896.58 nm in 22 °C. Application of NTB resulted in microspheres of rather smaller size, 332.48 nm in 22 °C. As a result of replacement of the cross-linking agent MBA with PEG-DMA, there was also a significant reduction in the D_H_, leading to a size of 100.69 nm. The differences in the D_H_ as well as the differences in the release rate may be ascribed to the functional groups implemented to the polymeric particles in the course of SFPP. The respective co-monomers and used crosslinkers are presented in the following image (Figure 4).

It was found that the initiator did not affect the particle size. The use of a hydrophilic co-monomer, a PEG-MA, which had a long chain with ethoxy groups, produced relatively high D_H_. After crossing the VPTT, the chains probably adhere to the surface of the microspheres, which caused a large reduction of D_H_. The lipophilic co-monomer NTB generates branched chains. After crossing the VPTT there was a drop in the value of D_H_, but not as large as in the case of F2. Chains of NTB were not adhering so strongly to the surface of the microspheres because of their branching, as was the case for microspheres with a hydrophilic co-monomer.

**Figure 5:**
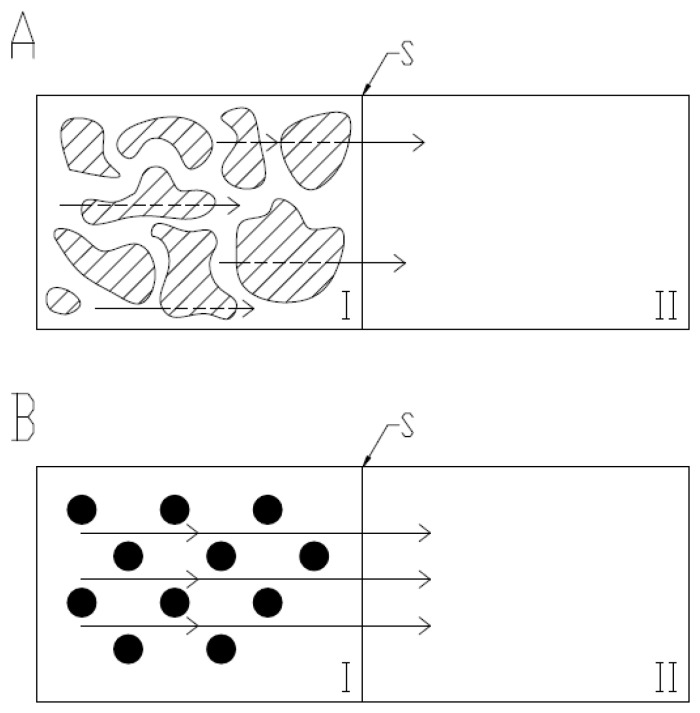
The schematic representation of naproxen diffusion (arrows) from donor compartment (I) to acceptor compartment (II) via semipermeable membrane at 22 °C (**A**) and 42 °C (**B**). The hatched region represents the polymer particles in expanded state, whereas the black dots represent the shrunken polymer.

The release of NS from hydrogels based on thermosensitive NIPA derivatives and HPMC is presented on Figure 2A–D, where both courses of release are presented for each hydrogel: at 22 °C ± 0.5 °C and at 42 °C ± 0.5 °C. The process was rather complex. We observed a drastic change in release profile, as a result of temperature changes. The release of NS above LCST was much faster compared to the release rates at the temperature below VPTT. This may be ascribed to the fact of the contraction of the surface of the microspheres. At a lower temperature, the polymer is swelled, which results in a longer time of release for the drug, which must diffuse via viscous polymeric environment. At higher temperatures, the lattice structure of the polymer is disrupted and it has lower tendency to absorb water, so the drug penetrates via semipermeable membrane mostly from aqueous space between the particles of the polymer [25,26,27]. The proposed schematic diffusion in both environments is presented in Figure 5. The effect may be enhanced by ion dipole interaction between the metal cation and the amide, which could form a metal ion–amide complex and decrease the releasing rate [28].

It has been proven that the best mathematical model to demonstrate the kinetics of the drug release was the Higuchi model in most cases. Various mathematical models are used for description of drug release from polymeric matrices [29,30]. In our research all evaluated formulations (FP1–FP4) showed good correlation to the Higuchi model, particularly in the release process at higher temperature of 42 °C [3,30]. The results of linear regression analysis are also shown in Table 2 and Table 3. Comparing the preparations NIPA derivatives (F1–F4) and the control formulation with HPMC, it can be seen that the addition of thermo-sensitive polymer of NIPA modified the release process of NS, and the influence of NIPA derivatives on drug release was observed in other studies performed by various researchers [5,24,31,32]. The release process does not reflect huge differences between preparations FP1–FP4, but there are some deviations that may be caused by the presence of specific functional groups derived from additional monomers or crosslinking agents. An increased release of NS from FP4 was the most emphatic compared to FP1–FP3. The increase may be ascribed to the presence of the crosslinking agent PEG-DMA, and the high values of standard deviations may indicate complex influence of the hydrophilic polyoxyethylene group on the release pattern on NS from the nanoparticles. Preparation FP3 has a very similar release course of NS, compared to the reference formulation with HPMC only, both at 22 °C and 42 °C, but the high variability of released amounts of NS in the FP3 batch hinders direct inferences about the intermolecular effects in the hydrogel. The high variability of the release data in the sample FP3 may result from the presence of hydrophilic and lipophilic chains interacting in the sample, and will further investigated. In formulation FP4 there was the greatest difference in the release of NS between 22 °C and 42 °C. The slowest release of NS from the polymer matrix was observed in the preparation FP2 at 22 °C. In the case of high value of VPTT, we observed fast NS release, as it was in the case of FP4.

## 4. Experimental Section

### 4.1. Materials

Naproxen sodium (NS, (*S*)-(+)-2-(6-Methoxy-2-naphthyl)propionic sodium conforming to USP standards (Sigma Aldrich, Sternheim, Germany), hydroxypropyl methyl cellulose (HPMC, viscosity of 2600–5600 cP, 2% in H_2_O at 20 °C (Sigma Aldrich, Ashland, Wilmington, DE, USA), *N*-isopropyl acrylamide (NIPA, 97%, Sigma Aldrich, Sternheim, Germany), *N*-tertbutyl acrylamide (NTB, 99%, Acros Organics, Geel, Belgium), *N*,*N*′-methylene bisacrylamide (MBA, 99 %, Sigma Aldrich, Sternheim, Germany), methyl ether-acrylate poly(ethylene glycol) with nine oxyethylene groups (PEG-MA, Mw = 480 Da, 99%, Sigma Aldrich, Sternheim, Germany), poly(ethylene glycol) dimethacrylate (PEG-DMA, Mw = 2000, Sigma Aldrich, Sternheim, Germany), dihydrochloride 2,2′-azobis (2-methyl propionamidine) (ABAP, granules, 97%, Sigma Aldrich, Sternheim, Germany) were obtained from commercial and industrial suppliers and used without further purification. The dialysis membrane with a molecular mass cutoff (MWCO) of 12.000–14.000 Da was obtained from the company Visking Medicell International Ltd. (London, UK). Deionized water was obtained from the ionic column according to monography of the purified water from the European Pharmacopoeia, and used in all studies. Dimethylsulfoxide (DMSO-*d*_6_) used for NMR spectrometry was obtained from Euriso-Top (Sigma Aldrich, St. Aubin, France).

### 4.2. Synthesis of the Polymers

NIPA-based polymers were synthesized using the SFPP method, without any emulsifier. The properly prepared reactor was used to complete the synthesis. The 2.0 liter glas reactor flask with three-necked easily detachable glas cover was applied. It was filled with 600 g of deionized water previously heated to 70 °C. The stable temperature in reactor was maintained using water bath with feedback-control of temperature via temperature sensor immersed in the reacting fluid, and connected to the heat plate. The initiator was previously dissolved in 150 g of deionized water and added to the reactor. After stabilizing the temperature of the reaction, suitable combinations of co-monomers in 250 g of deionized water were prepared and delivered into the reactor flask. The composition of the individual samples are presented in the Table 4. The reactor was connected to the Allihn reflux condenser. The 150 rpm velocity was used for teflon-coated magnetic stirrer immersed in the reactant fluid through the entire process.

All received formulations were purified by dialysis. The process consisted of measuring the electrical conductivity every 24 h in the compartment acceptor. After each measurement the external solution was exchanged. The purification process was completed when the measured conductivity had a value of less than 1 µS·cm^−1^. Then all the formulations were freeze-dried, to increase their stability and to enable their use in further studies. Steris freeze drier Lyovac GT2 was applied for freez-drying.

### 4.3. NMR Evaluations

NMR spectra were measured for all used monomers and obtained polymers using the Bruker (Faellanden, Switzerland) 300 MHz NMR device in the Integrated Education and Innovation Centre of the Faculty of Pharmacy at the Medical University in Wroclaw. Measurements were carried at 24 °C. 5 mg of obtained polymer, previously dried, was used for every analysis. 0.8 mL of product was dissolved in DMSO. All samples were soluble in DMSO, centrifugation or filtration were not necessary.

### 4.4. Evaluation of Hydrodynamic Diameter and VPTT

The D_H_ of the obtained polymer was measured by using a Zeta Sizer Nano Malvern Instruments (Malvern Instruments, Malvern, UK), via dynamic light scattering (DLS) at a wavelength of 678 nm. The study used an aqueous dispersions of the synthesized polymers, samples were diluted ten-fold with deionized water, and then filtered through a Whatman nano-filter 0.2 μm PVDF. They were subjected to evaluation using a 173° backscattering measurement system, and compared to the parameters of the Mark-Houwink constants. Each test was repeated five times, then they were treated in the software Zeta Sizer Nano Software version 5.03 (Malvern Instruments, Malvern, UK).

### 4.5. Preparation of Hydrogels with Naproxen Sodium

Hydrogel formulations F1–F4 of NS were prepared in a specific way ex tempore. Each of them contained 0.4 g NS, which was sprinkled on the surface of distilled water. Then, 0.5 g of the synthesized polymer F1–F4 was used, previously dissolved in the water. Afterwards 0.5 g of HPMC was added to the solution, the whole was thoroughly mixed and homogenized. Prepared samples were stored at room temperature for 24 h. In a further step they were used in release tests at different temperatures. The composition of the tested hydrogels is presented in the Table 5.

### 4.6. Drug Release from Thermosensitive Hydrogels

The process of release of NS from the hydrogels was performed with techniques used for testing the transdermal therapeutic systems according to Ph. Eur. using Erweka equipment (Erweka, Heusenstamm, Germany). The dialysis container was placed in a thermostated vessel, containing 1000 mL of double-distilled water (liquid acceptor) with a speed of 50 rpm. The amount of diffused NS was a measure of the release rate. The processing temperature was kept between 22 ± 0.5 °C and 42 ± 0.5 °C. The measurement was carried out for 2 h. Each sample was subjected to two analyzes, in separate reaction vessels. In certain time intervals 1.5 mL of the solution was pulled out to measure the amount of released drug. The concentration of released substance was measured spectrophotometrically at a wavelength of 298 nm by reading absorbance. The UV-VIS spectrophotometer V-530 (Jasco, Tokyo, Japan) was used to carry out the test. Mathematical models were used: zero order, first and second order and Higuchi equation to evaluate the kinetics of drug release from hydrogels based on thermo-sensitive polymer. The values obtained from in vitro studies of drug release were used to calculate the correlation coefficient.

## 5. Conclusions

Application of cationic initiator ABAP enables SFPP with the use of hydrophilic and lipophilic co-monomers PEG-MA and NTB, respectively. The resulting NIPA derivatives are nanosized in the range of 100.69–1117.60 nm depending on the components used and temperature of measurements. Modification of the cross-linker type influences the properties of synthesized polymeric particles. The NIPA derivatives obtained via SFPP may be formulated as hydrogel preparations, using HPMC. The obtained formulations present varied half-release times, depending on the type of applied NIPA derivatives in hydrogel formulations. At 18 °C the release rates are lower compared to the reference HPMC hydrogel, whereas at 42 °C the release rates are significantly higher. The synthesized thermosensitive polymers enable temperature-triggered release of NS in in vitro conditions.

## Figures and Tables

**Table 1:** Hydrodynamic diameter (D_H_) and volume phase transition temperature (VPTT) values of the synthesized polymers.

Polymer	D_H_ 22 °C (nm)	SD	D_H_ 42 °C (nm)	SD	VPTT (°C)
F1	798.48	9.31	413.18	3.79	35
F2	896.58	2.12	343.96	4.48	•
F3	332.48	6.94	100.69	0.44	26
F4	155.46	2.75	163.62	6.22	•

F1–F4: synthesized polymers, •: there were no changes observed in the D_H_ value, SD—standard deviation.

**Table 2:** The release rates K_ZO_, K_FO_, K_SO_, K_H_, and correlation coefficients (r^2^), respectively, for the evaluated kinetic models: zero order process (ZO), first order process (FO), second order process (SO), and Higuchi model (H) for the release of naproxen sodium from formulations FP1–FP4, at temperature of 22 °C.

Model	Parameter	Formulation									
		FP1		FP2		FP3		FP4		HPMC	
		Value	SD	Value	SD	Value	SD	Value	SD	Value	SD
ZO	K_ZO_ (% × min^−1^)	5.18 × 10^−2^	3.76 × 10^−3^	5.08 × 10^−2^	4.94 × 10^−4^	5.42 × 10^−2^	8.34 × 10^−3^	5.64 × 10^−2^	4.69 × 10^−3^	5.86 × 10^−2^	1.86 × 10^−3^
	r^2^	0.99488	0.00377	0.99661	0.00030	0.99150	0.00251	0.99129	0.00014	0.99367	0.00099
FO	K_FO_ (min^−1^)	5.59 × 10^−4^	4.31 × 10^−5^	5.46 × 10^−4^	2.13 × 10^−6^	5.87 × 10^−4^	9.88 × 10^−5^	6.11 × 10^−4^	5.51 × 10^−5^	6.34 × 10^−4^	2.05 × 10^−5^
	r^2^	0.99543	0.00353	0.99697	0.00006	0.99261	0.00214	0.99248	0.00022	0.99471	0.00094
SO	K_SO_ (min^−1^ × %^−1^)	6.04 × 10^−6^	4.93 × 10^−7^	5.88 × 10^−6^	1.12 × 10^−8^	6.37 × 10^−6^	1.17 × 10^−6^	6.62 × 10^−6^	6.44 × 10^−7^	6.86 × 10^−6^	2.26 × 10^−7^
	r^2^	0.99591	0.00327	0.99727	0.00366	0.99363	0.00179	0.99358	0.00029	0.99566	0.00087
H	K_H_ (min^0.5^)	7.82 × 10^−1^	5.28 × 10^−2^	7.64 × 10^−1^	1.05 × 10^−2^	8.25 × 10^−1^	1.29 × 10^−1^	8.59 × 10^−1^	7.12 × 10^−2^	8.90 × 10^−1^	2.71 × 10^−2^
	r^2^	0.99540	0.00131	0.99367	0.00366	0.99940	0.00038	0.99977	0.00012	0.99954	0.00023
BF		SO		SO		H		H		H	

SD—standard deviation, BF—best fit.

**Table 3:** The release rates K_ZO_, K_FO_, K_SO_, K_H_, and correlation coefficients (r^2^), respectively, for the evaluated kinetic models: zero order process (ZO), first order process (FO), second order process (SO), and Higuchi model (H) for the release of naproxen sodium from formulations FP1–FP4, at temperature of 42 °C.

Model	Parameter	Formulation									
		FP1		FP2		FP3		FP4		HPMC	
		Value	SD	Value	SD	Value	SD	Value	SD	Value	SD
ZO	K_ZO_ (% × min^−^^1^)	7.59 × 10^−2^	1.47 × 10^−3^	7.46 × 10^−2^	6.83 × 10^−5^	6.81 × 10^−2^	4.87 × 10^−3^	8.21 × 10^−2^	1.12 × 10^−2^	5.93 × 10^−2^	3.08 × 10^−3^
	r^2^	0.99518	0.00166	0.99278	0.00252	0.99065	0.00080	0.98943	0.00210	0.98835	0.00110
FO	K_FO_ (min^−1^)	8.38 × 10^−4^	1.29 × 10^−5^	8.20 × 10^−4^	2.31 × 10^−6^	7.48 × 10^−4^	6.20 × 10^−5^	9.18 × 10^−4^	1.44 × 10^−4^	6.46 × 10^−4^	3.91 × 10^−5^
	r^2^	0.99629	0.00143	0.99396	0.00258	0.99215	0.00060	0.99138	0.00162	0.98984	0.00094
SO	K_SO_ (min^−1^ × %^−1^)	9.25 × 10^−6^	1.07 × 10^−7^	9.03 × 10^−6^	5.91 × 10^−8^	8.22 × 10^−6^	7.75 × 10^−7^	1.03 × 10^−5^	1.82 × 10^−6^	7.03 × 10^−6^	4.86 × 10^−7^
	r^2^	0.99725	0.00121	0.99500	0.00262	0.99352	0.00043	0.99314	0.00119	0.99123	0.00080
H	K_H_ (min^0.5^)	1.15	2.48 × 10^−2^	1.13	8.79 × 10^−4^	1.04	7.51 × 10^−2^	1.25	1.74 × 10^−1^	9.05 × 10^−1^	4.80 × 10^−2^
	r^2^	0.99846	0.00056	0.99763	0.00239	0.99987	0.00009	0.99983	0.00013	0.99984	0.00013
BF		H		H		H		H		H	

SD—standard deviation, BF—best fit.

**Table 4:** Substrate compositions of the prepared polymers.

Substrates (*w*/*w*)	Type of Component							
	NIPA		Cross-Linker		Co-Monomer		Cationic Initiator	Solvent Water
			MBA	PEG-DMA	PEG-MA	NTB	ABAP	
Type of polymer	F1	0.5	0.05	-	-	-	0.05	99.40
	F2	0.5	0.05	-	0.05	-	0.05	99.35
	F3	0.5	0.05	-	-	0.05	0.05	99.35
	F4	0.5	-	0.05	-	-	0.05	99.40

NIPA: *N*-isopropyl acrylamide, MBA *N*,*N*′-methylene bisacrylamide, PEG-DMA poly(ethylene glycol) dimethacrylate, PEG-MA methyl ether-acrylate poly(ethylene glycol), NTB *N*-tertbutyl acrylamide, ABAP dihydrochloride 2,2′-azobis (2-methyl propionamidine).

**Table 5:** Compositions of formulations FP1–FP4 with the synthesized polymers.

Composition%	Formulation				
	FP1	FP2	FP3	FP4	REF
NS	4.0	4.0	4.0	4.0	4.0
F1	0.5				
F2		0.5			
F3			0.5		
F4				0.5	
HPMC	0.5	0.5	0.5	0.5	0.5
H_2_O	95.0	95.0	95.0	95.0	95.5

NS: naproxen sodium, F1–F4: polymers synthesized by SFPP according to the Table 4, HPMC: hydroxypropyl methyl cellulose.
